# Small-volume *in vitro* lipid digestion measurements for assessing drug dissolution in lipid-based formulations using SAXS

**DOI:** 10.1016/j.ijpx.2022.100113

**Published:** 2022-02-09

**Authors:** Nafia F. Khan, Malinda Salim, Syaza Y. Binte Abu Bakar, Kurt Ristroph, Robert K. Prud'homme, Adrian Hawley, Ben J. Boyd, Andrew J. Clulow

**Affiliations:** a UCL School of Pharmacy*,* University College London, 29-39 Brunswick Square*,* London WC1N 1AX*,* UK; b Drug Delivery, Disposition and Dynamics*,* Monash Institute of Pharmaceutical Sciences*,* Monash University (Parkville Campus), 381 Royal Parade*,* Parkville, Victoria *3052,* Australia; c Department of Chemical and Biological Engineering*,* Princeton University*,* Princeton*,* NJ *08544,* United States; d Australian Synchrotron, ANSTO, 800 Blackburn Road*,* Clayton*,* Victoria *3168,* Australia; e Department of Pharmacy*,* University of Copenhagen*,* Universitetsparken 2*, 2100* Copenhagen*,* Denmark

**Keywords:** Lipid digestion, Lipid-based formulations, Infant formula, Small angle X-ray scattering, LBFs, lipid-based formulations, FA, fatty acid, SAXS, small-angle X-ray scattering, IF, infant formula, CFZ, clofazimine, FNP, Flash NanoPrecipitation, Hf, halofantrine, FFA, free fatty acid, THF, tetrahydrofuran, PLA, polylactic acid, CIJ, confined impinging jet, PDI, polydispersity index, DLS, dynamic light scattering, LC, loading capacity, EE, encapsulation efficiency, iFast, simulated infant fasted intestinal media, XRD, X-ray diffraction, F X, polymorphic form X, API, active pharmaceutical ingredient, 3DP, 3D-printed

## Abstract

Lipid-based formulations improve the absorption capacity of poorly-water-soluble drugs and digestion of the formulation is a critical step in that absorption process. A recent approach to understanding the propensity for drug to dissolve in digesting lipid-based formulations couples an *in vitro* pH-stat lipolysis model to small-angle X-ray scattering (SAXS) by means of a flow-through capillary. However, the conventional pH-stat apparatus used to measure the extent of lipid digestion during such experiments requires digest volumes of 15–30 mL and drug doses of 50–200 mg, which is problematic for scarce compounds and can require excessive amounts of formulation reagents. This manuscript describes an approach to reduce the amount of material required for *in vitro* lipolysis experiments coupled to SAXS, for use in instances where the amount of drug or formulation medium is limited. Importantly, this was achieved while maintaining the pH stat conditions, which is critical for maintaining biorelevance and driving digestion to completion. The digestibility of infant formula with the poorly-water-soluble drugs halofantrine and clofazimine dispersed into it was measured as an exemplar paediatric-friendly lipid formulation. Halofantrine was incorporated in its powdered free base form and clofazimine was incorporated both as unformulated drug powder and as drug in nanoparticulate form prepared using Flash NanoPrecipitation. The fraction of triglyceride digested was found to be independent of vessel size and the incorporation of drug. The dissolution of the two forms of clofazimine during the digestion of infant formula were then measured using synchrotron SAXS, which revealed complete and partial solubilisation over 30 min of digestion for the powdered drug and nanoparticle formulations, respectively. The main challenge in reducing the volume of the measurements was in ensuring that thorough mixing was occurring in the smaller digestion vessel to provide uniform sampling of the dispersion medium.

## Graphical abstract

**Figure:**
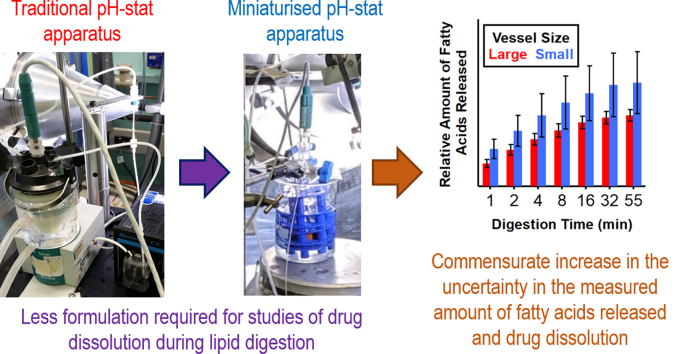

## 1 Introduction

Drug candidates emerging from discovery programs are often highly potent but poorly water-soluble (Porter et al., 2007). This presents challenges during drug development as a reduced systemic exposure often results following oral drug delivery. For lipophilic poorly water-soluble drugs, the inclusion of natural or synthetic lipids and lipophilic excipients within these formulations can enhance oral bioavailability. Lipid-based formulations (LBFs) are intended to maintain a lipophilic drug in a dissolved state throughout gastrointestinal transit and must overcome changes in drug solubility during lipid digestion to achieve drug delivery through the intestine (Khan et al., 2016a). Digestion therefore represents a critical step in lipid-based drug delivery as lipases, in particular human pancreatic lipase, hydrolyse triacylglycerols (TAGs) in formulations to produce more polar lipid species, 2-monoacylglycerols (2-MAGs) and fatty acids (FAs) (Jannin et al., 2015; Bakala N'Goma et al., 2012). These amphiphilic digestion products can self-assemble into lyotropic liquid crystalline structures in the small intestine (Salentinig et al., 2013, Salentinig et al., 2015; Clulow et al., 2018; Pham et al., 2020), in turn these structures interact with endogenous amphiphilic molecules such as bile acids to form colloidal mixed micelles with a variety of structures (Hjelm et al., 2000; Hjelm et al., 1995; Pabois et al., 2021; Rezhdo et al., 2017; Gustafsson et al., 1999; Clulow et al., 2017). The digestion products are also capable of solubilising poorly water-soluble drugs dispersed in the LBF (Shrestha et al., 2014; Salim et al., 2019a; Boyd et al., 2018; Salim et al., 2020a) making them available for subsequent absorption (Tan et al., 2010; Borné et al., 2002; Boyd et al., 2007).

Milk and infant formula (IF) are oil-in-water emulsions that can be used as a LBF that is particularly suited to paediatric populations (Lindmark Månsson, 2008). About 98% of lipids in full cream milk (3–5% *w/v* fat) are TAGs, present as globules emulsified in the aqueous phase (Jensen, 2002). Macheras and co-workers previously used milk in solubility and bioavailability studies to demonstrate increased absorption of poorly-water-soluble drugs (Macheras and Reppas, 1986; Soulele and Macheras, 2015; Kytariolos et al., 2013). However, these studies did not consider the effect of lipid digestion but measured the dissolution properties of drugs into the fat globules and casein micelles in undigested milk. Given that lipophilic drugs may partition differently into milk lipids before and after digestion, it is important to understand the changes in drug solubility during lipid digestion to best identify suitable milk-based formulations (Boyd et al., 2018; Salim et al., 2020b). Another approach to enhancing drug solubility and dissolution is the use of drug nanoparticle formulations. The small size of the drug particles increases the total effective surface area to facilitate more rapid dissolution while the presence of amphiphilic excipients and potential changes to the solid state of the drug upon conversion to nanoparticles can also improve solubility (Müller et al., 2001). One example of such a nanoparticle formulation is for clofazimine (Feng et al., 2018, Zhang et al., 2017), an antibiotic used to treat leprosy and cryptosporidiosis (Browne and Hogerzeil, 1962; Love et al., 2017). When prepared by Flash NanoPrecipitation (FNP), clofazimine showed more rapid dissolution during digestion in milk and IF compared to the crystalline starting drug material (Salim et al., 2019a).

*In vitro* lipolysis experiments using a pH-stat to maintain a fixed pH during liberation of fatty acids is a typical approach to follow the kinetics of digestion of lipids in a formulation. Samples are often retrieved for offline drug assay by chromatography; however, this requires inhibition of the lipase and a separation step to discriminate solubilised drug from solid undissolved drug (Stillhart et al., 2013; Salim et al., 2018; Salim et al., 2019b). One approach to circumvent the uncertainty involved in the “sample and separate” method is to study the process of solubilisation *in situ*. A recent review highlighted the use of ion-selective electrodes, in-line UV probes, Raman scattering spectroscopy, and synchrotron X-ray scattering, for the real time analyses of drug solubilisation and precipitation in digesting lipids (Kuentz, 2019). On a structural level, low frequency Raman scattering spectroscopy and X-ray scattering are uniquely placed to evaluate the dissolution/precipitation of crystalline drug and any polymorphic changes occurring during digestion of lipid formulations (Salim et al., 2019a; Salim et al., 2018; Salim et al., 2019c; Salim et al., 2020c; Boyd et al., 2018).

The coupling of *in vitro* lipid digestion with *in situ* small angle X-ray scattering (SAXS) has emerged as a powerful tool to predict the behaviour of drugs and their distribution into various self-assembled lipid structures. SAXS can be used to characterise changes in the mesophases of lipid-based drug delivery systems under stimuli including changes in pH (Li et al., 2019; Mertins et al., 2020; Fong et al., 2020), changes in temperature (Tran et al., 2018; Fong et al., 2009, Fong et al., 2014), exposure to endogenous surfactants and cells (Fornasier et al., 2021; Bor et al., 2022), and digestion (Clulow et al., 2018, Salentinig et al., 2013, Salentinig et al., 2015; Salim et al., 2019c; Warren et al., 2011; Khan et al., 2016b; Salvati Manni et al., 2021; Thorn et al., 2020). The use of a high-flux synchrotron X-ray source enables a time-resolved method to study these dynamic transitions between liquid crystalline structures during digestion (Boyd and Clulow, 2021). Concurrently, the presence of crystalline drug can also be directly monitored through X-ray diffraction at wider angles. Characteristic diffraction peaks can be identified and attributed to the presence of, and more specifically, the disappearance of drug if it is solubilised during digestion by tracking the change in peak area over time. In addition, the approach also allows the identification of particular polymorphic forms of the drug, allowing additional information on polymorphic transformations during digestion to be elucidated. The traditional method for coupling *in vitro* lipolysis model to SAXS is to circulate a portion of the sample through a capillary located in the X-ray beam (Warren et al., 2011). This method requires reasonable volumes of sample (15–30 mL) per measurement because of the need for simultaneous pH monitoring under stirring/mixing in the vessel and filling of the dead volume of the flow loop through the peristaltic pump and capillary. This can be an unreasonably large volume when studying samples containing either drug substances that are in short supply, especially during early-stage drug discovery, or in the case where excipients in the lipid formulation or the digestion medium are expensive or limited in availability, such as bile salts or in the study of drugs in human milk, respectively.

Some studies have attempted to use a fixed low volume digestion format without dynamic pH control, such as a 96 well plate or a fixed capillary not in flow-through and without dynamic pH control (Wadsäter et al., 2018; Mosgaard et al., 2017). The benefits of this method include sample volumes on the order of hundreds of microlitres, simple sample preparation and loading into the plate/capillary. There are also advantages to the automated presentation of large numbers of samples into the synchrotron X-ray beam. However, there are a number of problems that arise with this method. There is a need for extremely high and unrealistic buffer concentrations, which in our experience are still not sufficient to maintain a constant pH and also influences the electrostatic interactions between charged lipids in the system through high ionic strength. There is also a disconnect between the initiation of digestion and the scattering measurements through the time required to load the samples and introduce them to the beam, which makes this approach more amenable to measurements on the final products of digestion rather than intermediates. Furthermore, a lack of stirring in well plates/capillaries can lead to segregation and inhomogeneous samples with consequent uncertainty about how representative the measurement is of the whole system.

**Fig. 1:**
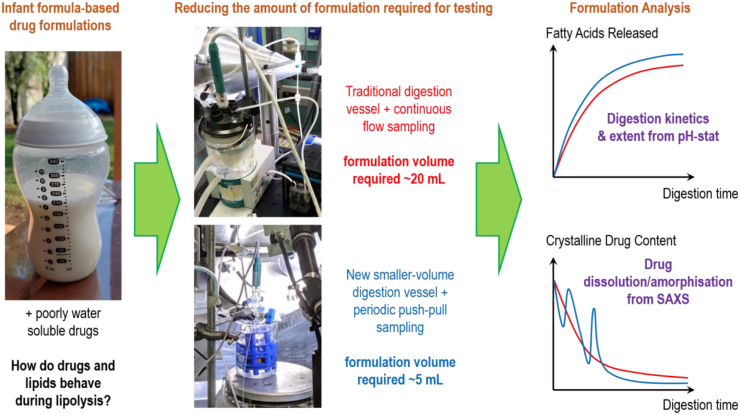
Schematic figure illustrating the overall concept of the study focussing on evaluating the alternative small-volume *in situ* digestion configuration using clofazimine and halofantrine in infant formula as model systems.

In this study we therefore examined the possibility of using a small-volume *in vitro* lipolysis apparatus coupled to SAXS that provides an avenue to minimise material consumption when either drug or formulation components are in limited supply, whilst maintaining pH control. The sample volume could be reduced to 5 mL and the drug dose proportionally reduced by reducing the volume of the digestion vessel and modifying the method of introducing the sample into the X-ray beam. The poorly-water-soluble drugs, halofantrine (Hf) and clofazimine (CFZ), were used as model drugs digested in infant formula (Fig. 1), scaling down the previously described studies with these drugs in *in vitro* lipid digestion experiments coupled to SAXS (Salim et al., 2019a; Boyd et al., 2018). Halofantrine free base and clofazimine were incorporated into formulations using the starting crystalline drug material. Clofazimine was also dispersed in both free base and nanoparticle form, with the aim of directly comparing results with the small-volume *in vitro* lipolysis model and the conventional *in vitro* digestion apparatus, herein referred to as the “large-volume” apparatus. IF powder was reconstituted with water to 3.8% *w/v* fat to be used as the lipid formulation for drugs. SAXS was used as a time-resolved method to observe the dynamic changes in the liquid crystalline structures of digestion products and to establish the drug solubilisation profiles during digestion.

## 2 Experimental methods

### 2.1 Materials

Hf free base (>99% purity) was purchased from GlaxoSmithKline (King of Prussia, PA). CFZ free base (≥98% purity), trizma maleate reagent grade, sodium azide (≥99%, Fluka), mannitol, sodium taurodeoxycholate hydrate (NaTDC) and tetrahydrofuran (THF) were purchased from Sigma-Aldrich Pty. Ltd. (MO, U.S.A.). Calcium chloride dihydrate (>99% purity), sodium hydroxide pellets (minimum 97% purity), and salicylic acid were purchased from Ajax Finechem (New South Wales, Australia). Hydrochloric acid (aqueous solution) was purchased from LabServ (Ireland). Sodium chloride (>99% purity) was purchased from Chem Supply (South Australia, Australia). Polylactic acid (PLA) filament was purchased from a local supplier, Imaginables Pty Ltd. (Victoria, Australia). 1,2-dioleyl-*sn*-glycero-3-phosphatidylcholine (DOPC) was purchased from Cayman Chemical (MI, U.S.A.). Soy lecithin (refined grade) was purchased from MP Biomedicals (OH, U.S.A.). Powdered infant formula (IF) was kindly donated by Medicines for Malaria Venture (MMV, Switzerland) and its nutritional information is detailed elsewhere (Pham et al., 2020). Water was sourced from Milli-Q-Millipore purification systems (Merck Millipore, Australia) at the point of use. Lipase (USP-grade pancreatin extract) was purchased from Southern Biologicals (Victoria, Australia). To account for batch-to-batch variations in the lipase activity, a standard preparation method was followed (Khan et al., 2016a). The aqueous-soluble components of lipase were extracted by repeated centrifugation of pancreatin dispersions in water (2205 ×*g* for 15 min) to remove most of the insoluble components of pancreatin. The supernatant was freeze-dried to produce powdered lipase. The lipolytic activity of this powder was then determined using a standard tributyrin assay preceding digestion measurements, with observed activities of 500–800 TBU/mL. The test was performed by digesting 6 g of tributyrin in 18 mL of tris buffer (pH 7.5, 150 mM sodium chloride, 5 mM calcium chloride dihydrate, 6 mM sodium azide) with 2 mL of redispersed lipase powder in the same buffer (217 mg of lipase powder were reconstituted per 1 mL of buffer). Unless otherwise stated, all chemicals were used without further purification. Simulated infant fasted intestinal media (iFast) was prepared in tris buffer and contained 5.44 mM NaTDC and 1.13 mM DOPC (Kamstrup et al., 2017).

### 2.2 3D Printing of the sample holder for small-volume experiments

**Fig. 2:**
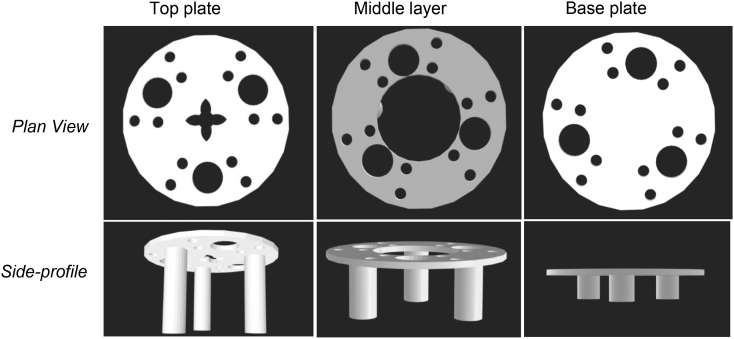
The 3D-printed holder for 20 mL glass scintillation vial inside a 250 mL beaker. From left to right are the top plate, inner layer(s) and base plate. Plan view and side-profiles are shown. Details on the dimensions of the various components can be found in Table S2 (electronic supporting information).

**Fig. 3:**
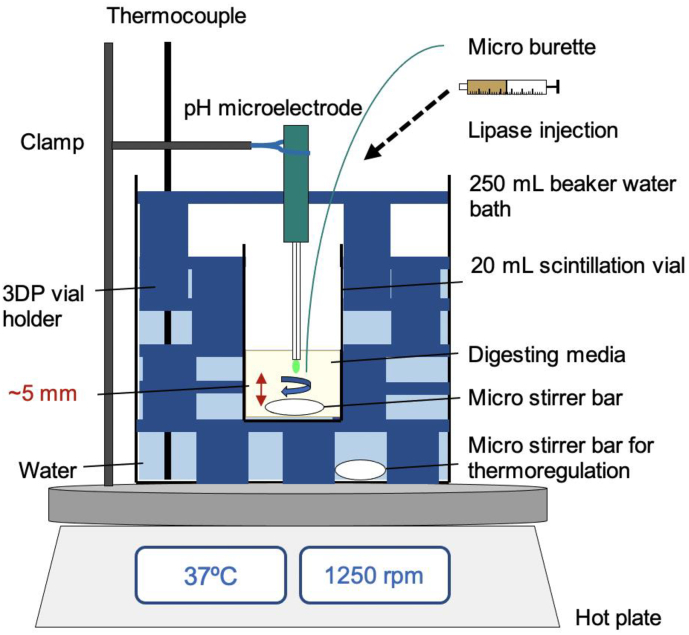
Schematic of the small-volume thermoregulated digestion vessel used in this work.

A vial holder was designed to hold a standard 20 mL scintillation vial in a 250 mL beaker to enable digestions on the scale of ~5 mL. The holder was fabricated from polylactic acid (PLA) polymer filament using a commercial fused-deposition modelling 3D printer, Ultimaker 2 (Ultimaker). Templates were designed using SketchUp Pro 2019 (Version 19.2.221). Three parts were produced to make the complete sample holder: a base plate on which a 20 mL scintillation vial sits but allows a stirrer bar beneath it to circulate thermostatted water at 37 °C around the vial, a middle section to support the vial and a top plate that sits at the top of the vial and controls the positioning of the pH probe, burette and tubing to allow the use of the pH-stat to control the pH during digestion (Fig. 2). Holes were included in all plates to fit a thermocouple for control of the temperature of the water surrounding the vial. Each plate had three legs separated by 120° close to the perimeter of the plate, corresponding holes displaced from the legs by 60° allow for the plates to be stacked on top of one another to build up the sample holder within a beaker. The diameter of all pieces was sufficient to fit inside a standard 250 mL beaker with the entire apparatus positioned on a thermocouple controlled hot plate with magnetic stirrer (Fig. 3).

### 2.3 Fabrication and characterisation of clofazimine nanoparticles

Clofazimine nanoparticles were prepared by the FNP process as described by Zhang et al. with a confined impinging jet (CIJ) mixer and some minor alterations to the reported procedure (Zhang et al., 2017). In brief, CFZ (50 mg) and soy lecithin (50 mg) were dissolved in THF (10 mL). An aliquot of this mixture (0.5 mL) and an equal volume of water (antisolvent) were rapidly mixed in a CIJ mixer and dispersed directly into 9 mL of water, decreasing the organic solvent fraction to 5% *w/v*. Mannitol (7.5 mg) was then added as a cryoprotectant (Feng et al., 2018) for the nanoparticles and 0.5 mL portions of the nanoparticle dispersions were frozen in dry ice for approximately 60 min. The samples were then lyophilised in a freeze-dryer, shelf temperature −40 °C, under vacuum (<1 mTorr) for 2 days before they were stored in a freezer at −20 °C.

The size distribution and polydispersity indices (PDIs) of the resulting particles were assessed by dynamic light scattering (DLS) using a Zetasizer Nano-ZS (Malvern Instruments, UK) equipped with a 4 mW, 633 nm laser, at a detection angle of 173°, at 25 °C in triplicate. For the determination of particle size, the viscosity of the dispersant was taken to be 0.89 cP when no THF was present (redispersed lyophilised particles) and 1.05 cP when THF (1.2 mol%) was present in the solutions prepared before lyophilisation (Aminabhavi and Gopalakrishna, 1995).

To determine the loading capacity (LC), a sample of the powdered nanoparticles (10 mg) was dissolved in THF (1 mL) and this solution was diluted 20-fold in methanol. The UV–visible absorbances of the resulting methanol solutions were measured at 450 nm on an EnSpire® Multimode UV plate reader (PerkinElmer, USA) and quantified based on a calibration curve of known clofazimine concentrations in the range 0–500 μg/mL.

(1) $$\mathrm{LC}\;\left(\%\right)=\frac{\mathrm{CFZ}\;\text{mass in}\;\mathrm{NPs}\;}{\text{total mass of}\;\mathrm{NPs}}\times 100$$

The loading capacity of the nanoparticles was used to determine the appropriate mass of nanoparticles to add to infant formula formulations to be equivalent to 12.5 mg of CFZ active pharmaceutical ingredient (API) as described below.

### 2.4 Formulation preparation

Halofantrine formulations were prepared firstly by mixing 10 mg Hf with 0.63 mL water and 63 μL HCl to simulate stomach conditions (gastric phase). Then, simulated infant fasted intestinal media (iFast, 1.25 mL), was added to the Hf sample to allow wettability of this highly lipophilic drug as previously reported (Boyd et al., 2018). Clofazimine formulations were prepared by mixing CFZ API (12.5 mg) or an appropriate amount of FNP powder to have an equivalent mass of CFZ in the dispersions with 0.71 mL water and 71 μL HCl to simulate the gastric phase. All mixtures were vortexed to thoroughly mix the drug with the acidic medium before dispersion in 5 mL IF.

### 2.5 Experimental configurations for large and small lipolysis experiments

**Large volume lipolysis experiments** were performed using the pH-stat method (902 STAT titration system, Metrohm AG, Herisau, Switzerland) as described previously (Zangenberg et al., 2001; Bolko et al., 2014). The samples were contained in an ~70 mL thermostatted glass vessel equipped with a magnetic stirrer bar and connected to a water bath to maintain the temperature at 37 °C. The pH of the samples was adjusted to 6.500 ± 0.005 with NaOH and HCl (0.2–5.0 M) to simulate the pH of the duodenum (Taylor, 1985) and a pH electrode was used to monitor the pH during digestions. Tiamo software (version 3.2, Metrohm, AG, Herisau, Switzerland) was used to monitor the addition of titrant and thereby the rate and extent of lipid digestion. Tris maleate buffer (50 mM trizma maleate, 150 mM sodium chloride, 5 mM calcium chloride, 6 mM calcium chloride dihydrate, pH 6.5) was used to reconstitute lyophilised lipase powder. After the addition of lipase to the formulation, lipolysis was allowed to proceed for 60 min and each digestion was carried out in triplicate. Some free fatty acids (FFAs) persist in the unionised state at pH 6.5 given the proximity of this pH to their pK_a_ values (Stillhart et al., 2014). To avoid underestimation of the total mol FFA liberated, a back-titration was performed at the end of digestion as previously described (Stillhart et al., 2014). In brief, at the end of the 60 min digestion period, the pH was rapidly increased to 9.0 using NaOH, for which the molar amount of NaOH required corresponds to the amount of unionised FAs released at the end of digestion. The full forward and back titrations were also performed with water as a control to determine titrant volumes added in the absence of lipolysis products. These volumes were subtracted from the total forward and back titration volumes for each sample to obtain the values of V(forward titration) and V(back titration), respectively. To rescale the forward titration volume and afford the total fatty acids released during digestion, the amount of titrant at each time point was multiplied by the following correction factor:

(2) $$\text{Correction factor}=\frac{V\left(\text{forward titration}\right)+V\left(\text{back titration}\right)}{V\left(\text{forward titration}\right)}$$

where V(forward titration) is the volume of NaOH added during the 60 min forward titration and V(back titration) is the volume of NaOH added during the back titration to pH 9 at the end of the 60 min digestion period.

All titration data are expressed as mean ± standard deviation (*n* = 3). Titration data were plotted and analysed using the IgorPro (version 8.0.4.2) graphing and analysis software. A one-way analysis of variance test (ANOVA) with Tukey *post hoc* analysis was applied to time points taken at 2.5, 5, 7.5, 10, 20 and 60 min. Differences between the amounts of titrant added to the different formulations or the same formulation in different types of digestion vessel were deemed to be statistically significant if *p* < 0.05.

**Small-volume lipolysis experiments** were conducted using the same pH-stat parameters as the large volume digestion experiments but with the small-volume sample holder shown in Fig. 3. A range of NaOH titrant concentrations from 0.2–2.0 M were trialled with the small-volume (5 mL) experiments with the aim to achieve less than 10% dilution of sample after 60 min digestion. Lower concentrations (0.2 and 0.4 M) provided up to 33% dilution of the digesting emulsions, whist 0.5 M NaOH gave ~8% dilution of formulation. This was also true for digestions performed in the large-volume experiment apparatus. Higher concentrations typically used in large-volume experiments (1.0 M and 2.0 M), were found to be too concentrated for the small-volume experiments, with a small-volume of titrant added creating too great an increase in pH for an accurate titration profile to be measured. Thus, 0.5 M NaOH was used as the titrant for all subsequent small- and large-volume lipid digestion experiments.

To determine the minimum volume that the small-volume digestion vessel could hold and still maintain pH monitoring, infant formula reconstituted without drug was used as the medium (infant formula powder containing 27.1 g fat/100 g powder was dispersed in water (5 mL) to a final fat content of 3.8% *w/v)*. The minimum volume was determined by initially adding 5 mL of dispersed IF to a 20 mL scintillation vial and then removing aliquots of IF using a syringe and tubing. IF was removed until the pH could no longer be reliably monitored, amounting to 0.8 mL (Fig. 3). Therefore, a minimum volume of 4.2 mL could be used with the small-volume digestion apparatus while maintaining the pH using the pH stat and with a gap of ~5 mm between the pH electrode and the magnetic stirrer bar. For all subsequent experiments, 5 mL IF (or water as a control) was used for each digestion. A 20 mL vial containing the IF/drug dispersions was held using the 3D-printed vial holder in a standard 250 mL beaker, using water as the heating medium (Fig. 3). A hotplate heated the water to 37 °C and the temperature was held constant under magnetic stirring, monitored with a thermocouple.

### 2.6 Synchrotron SAXS measurements coupled to *in vitro* digestion

To enable real-time monitoring of evolving lipid nanostructure and solubilisation of crystalline drug during digestion both the large- and small-volume apparatus were interfaced in slightly different ways with the SAXS beamline at the Australian Synchrotron (ANSTO, Clayton, Victoria) (Kirby et al., 2013). For large-volume samples, following pH adjustment to 6.500 ± 0.005, the formulation was pumped through a static 1.5 mm glass capillary at approximately 10 mL/min using a peristaltic pump, as described previously (Salim et al., 2019a; Warren et al., 2011). SAXS images were acquired using a Pilatus 2 M detector: 5 s acquisition time, 20 s delay with a sample-to-detector distance of 795 mm to afford a *q-*range of 0.02 < *q* < 1.94 Å^−1^ with a photon energy of 13.0 keV. Pancreatin dispersion was injected remotely using a syringe driver. The raw data were reduced to scattering profiles of *I*(*q*) *versus q* using the in-house developed software, ScatterBrain (version 2.82).

For the small-volume digestions, a new “push-pull” sample handling approach was utilised. The vial containing the drug/IF mixture was placed in close proximity to the quartz capillary fixed in the path of the X-ray beam. A short length of silicone tubing connected to the bottom of the capillary, was dipped into the sample to enable withdrawal of the digesting mixture into the capillary. Following pH adjustment to 6.500 ± 0.005, the sample (<800 μL) was aspirated at the desired time points from the digestion vessel into the capillary using an automated syringe driver. An automated sequence was used to move the syringe plunger, drawing the sample into the capillary for scattering measurements and then returning it to the vial once the scattering measurement was complete (hence, push-pull sampling). This avoids the dead volume of several mL unavoidable in the large volume configuration where the sample is circulated through a peristaltic pump.

### 2.7 X-ray diffraction measurements on clofazimine API and FNP powders

The polymorphic form of clofazimine nanoparticles prepared by FNP was measured and compared against the SAXS patterns of CFZ crystalline starting material and the different polymorphs previously observed in nanoparticles (Salim et al., 2019a). Solid powder samples were loaded into glass capillaries (1.5 mm outer diameter, Charles Supper, USA), which were placed in the synchrotron X-ray beam and scattering patterns were acquired for 1 s using the parameters described above.

## 3 Results

### 3.1 Characterisation of clofazimine nanoparticles

The clofazimine nanoparticles had a narrow particle size distribution with mean particle size 74.7 nm and polydispersity index 0.198 immediately after precipitation. However, following lyophilisation and reconstitution the particle size distribution became too broad to accurately determine a particle size by DLS, and the reported average particle size was much larger than those originally formed (Table S3). This is consistent with previously reported observations of the formation of lyophilised cakes of CFZ, surfactant and cryoprotectant during freeze-drying after FNP (Feng et al., 2018). The loading capacity of CFZ prepared by FNP was 22.5 wt% and therefore 55.6 mg of CFZ FNP powder was added to the infant formula formulation to be equivalent to 12.5 mg of CFZ API powder.

### 3.2 Kinetics and extent of digestion of infant formula-based drug formulations in the small- and large-volume measurement configurations

**Fig. 4:**
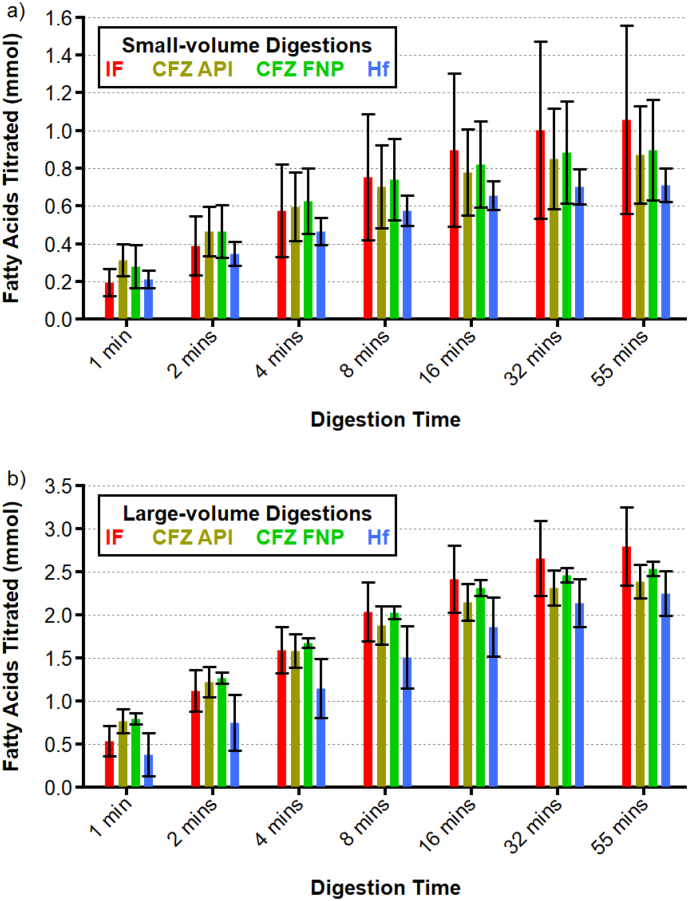
Titration profiles for infant formula (IF), infant formula with clofazimine pure drug (CFZ API), infant formula with clofazimine prepared by Flash NanoPrecipitation (CFZ FNP) and infant formula with halofantrine (Hf) (mean ± SD, *n* = 3) in (a) the small-volume apparatus using 5 mL IF and (b) the large-volume apparatus using 20 mL IF. Note that the x-axis scale is logarithmic (base 2) up to 32 min to emphasise the pseudo first order digestion kinetics in the early stages of digestion.

In this study, IF was dispersed in water, whereas previous experiments have utilised tris buffer as the dispersant (Dressman and Reppas, 2000, Salim et al., 2019a; Binte Abu Bakar et al., 2019). Here, it was found that formulations dispersed in water had sufficient buffer capacity for effective pH control and monitoring. Fig. 4 depicts titration profiles showing the amount of fatty acids titrated (with back-titration corrections) into IF and IF/drug dispersions during digestion in the small- and large-volume digestion vessels. Statistical comparisons of the amounts of fatty acids titrated in each digesting emulsion in either the small- or large-volume digestion vessels were performed by one-way ANOVA with Tukey *post hoc* analysis. Earlier time points (< 10 mins) were sampled more frequently since a large proportion of digestion takes place here (Tables S4-S6, electronic supporting information). In the early stages of digestion, the amount of fatty acids titrated followed a logarithmic increase. Each of the curves in Fig. 4 was fitted up to 32 min as a linear increase in fatty acids released *versus* the logarithm of time (base 2) and the corresponding gradients indicative of the relative rates of the digestions and Pearson correlation coefficients are given in Table S7 (electronic supporting information). After around half an hour of digestion, the titration curves began to plateau in both the small- and large-volume apparatus. The two formulations containing CFZ were digested at a similar rate throughout the digestions. On average, the initial amount of fatty acids released by the IF samples with no dispersed drugs was lower but the overall rate of digestion was faster and so by the end of the digestions the amount of fatty acids titrated were greater on average than the CFZ formulations. The amount of fatty acids titrated into the Hf formulations was consistently lower on average than the other three formulations throughout digestion.

No statistically significant differences (*p* < 0.05) were found between the amounts of fatty acids measured in the digesting IF in either the small- or large-volume apparatus at any stage during digestion (Tables S4 & S5, electronic supporting information). In the case of the small-volume apparatus, the relative uncertainties in the amounts of fatty acids titrated were greater than those in the large-volume apparatus. This was due to the variance in the volume of NaOH added during the back-titration to pH 9 used to fully ionise the liberated fatty acids at the end of the digestions. In the small-volume titrations, the variance in back titration volume was much greater than in the large-volume apparatus and this increased the corresponding uncertainty in the total volume of fatty acids titrated (Table S8, electronic supporting information). The uncertainty in the forward titration volumes measured using the small-volume apparatus were substantially lower than for the corresponding back titrations.

To more directly compare the titrations of the same formulation in either the small- or large-volume digestions, the amounts of fatty acids titrated in the large-volume digestions were divided by 4 (Fig. S4, electronic supporting information). Whilst there was a general trend for the amount of fatty acids titrated in the small-volume digestions to be greater on average than for the large-volume digestions, there were no statistically significant differences observed between the small- and large-volume titrations for any formulation at any time point (Table S6, electronic supporting information). Again, this was in part due to the greater uncertainty in the amounts of fatty acids titrated in the small-volume digestions, which primarily resulted from the larger variations in the volume of titrant added during the back titration (Table S8, electronic supporting information). Indeed, the total amount of fatty acid titrated in the forward titrations in the large-volume digestion vessel were around four times that in the small-volume digestion vessel in each case, as would be expected based on the relative change in sample volume. This further highlights that the discrepancy in total fatty acids titrated arises due to the larger variability in the back titration volume of the small-volume digestions, which will be elaborated on in the discussion section in light of the following results on measuring drug dissolution during digestion.

### 3.3 Clofazimine dissolution monitored by small angle X-ray scattering

**Fig. 5:**
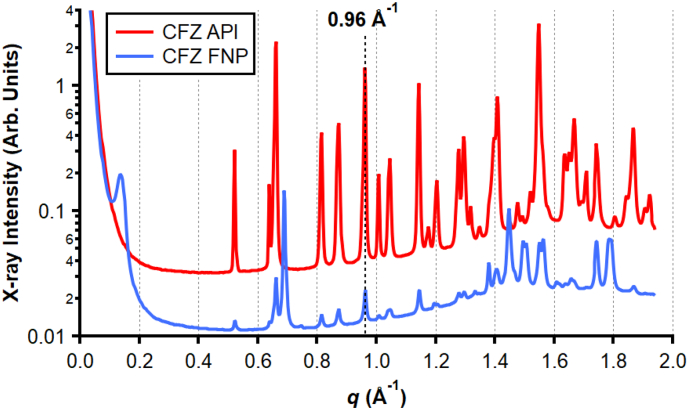
X-ray diffraction (XRD) profiles of clofazimine in glass capillaries for clofazimine pure drug (CFZ API), and clofazimine prepared by Flash NanoPrecipitation (CFZ FNP). The diffraction patterns are consistent with the previously reported clofazimine form I polymorph and the peak indicated at *q* = 0.96 Å^−1^ was subsequently used to monitor the presence of CFZ in digesting IF formulations (Bannigan et al., 2016). The diffraction profile for CFZ API has been offset in intensity for clarity by a factor of +0.7 (Arb. Units).

It has been shown previously using *in situ* synchrotron SAXS that clofazimine is not completely soluble in infant formula but that it dissolves or becomes amorphous as the lipids in infant formula are digested (Salim et al., 2019a). The CFZ API and CFZ FNP formulations were therefore used to confirm whether the small-volume digestion apparatus could be utilised for measuring the dissolution of lipophilic drugs. The diffraction patterns of the CFZ API and CFZ FNP powders were measured and are shown in Fig. 5. Characteristic peaks for the polymorphs of CFZ are observed at *q* = 0.96 Å^−1^ for the form I (F I) polymorph; *q* = 0.95 Å^−1^ for the form II (F II) polymorph; and *q* = 1.34 and 1.37 Å^−1^ for the form III (F III) polymorph (Bannigan et al., 2016). The diffraction patterns were consistent with the clofazimine F I polymorph being the major polymorph in both of the powders and the characteristic diffraction peak at *q* = 0.96 Å^−1^ was used to monitor the presence of crystalline drug in digesting infant formula (Salim et al., 2019a; Narang and Srivastava, 2002).

**Fig. 6:**
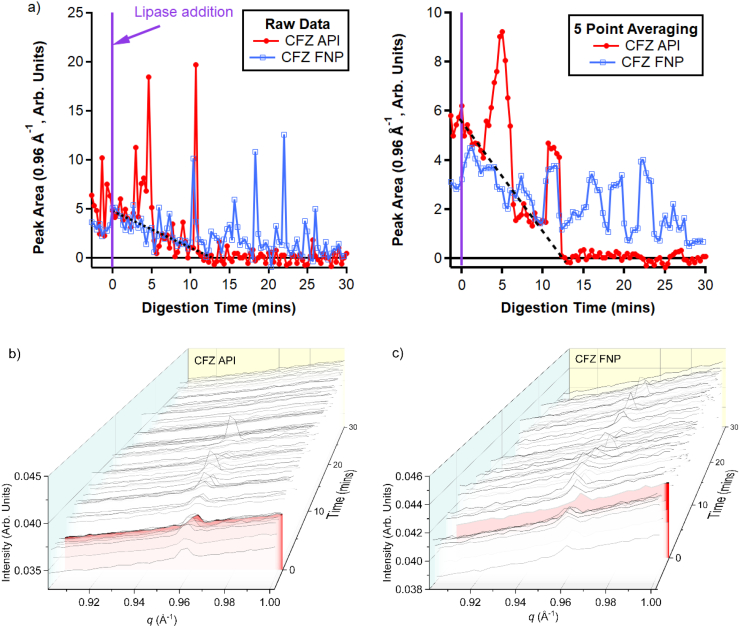
Dissolution of co-administered clofazimine in digesting infant formula. a) Residual integrated peak area of the diffraction peak consistent with clofazimine form I polymorph at *q* = 0.96 Å^−**1**^. Both the raw data and a plot with each data point representing the average of the previous five data points is shown. Data were collected from 5 mL dispersions of clofazimine pure drug (CFZ API) and clofazimine prepared by Flash NanoPrecipitation (CFZ FNP) powders in IF and the scattering data from which the peak areas are derived are shown in panels b) and c), respectively. The frame highlighted in red in panels b) and c) indicates the frame at time = 0 upon the injection of lipase. The dashed lines in the graphs in a) indicates the general reduction in the baseline amount of crystalline CFZ API in the formulations with increasing digestion time. (For interpretation of the references to colour in this figure legend, the reader is referred to the web version of this article.)

Formulations containing either CFZ API or CFZ FNP powder (12.5 mg equivalent mass of CFZ) in infant formula were digested and the scattering profiles of the formulations were monitored as a function of digestion time (Fig. 6). As the CFZ API formulation was digested, the area of the diffraction peak characteristic of CFZ F I generally reduced in intensity until there was little residual peak intensity after approximately 15 min of digestion, during which time most of the digestion of the infant formula lipids had taken place (Fig. 4). However, the intensity of the diffraction peak associated with CFZ F I was observed to sporadically increase in intensity up to 11 min of digestion in the CFZ API formulation, indicating that some residual crystalline clofazimine was present. The initial area of the CFZ F I diffraction peak was weaker in the formulation containing dispersed FNP powder, as it was in the pre-dispersed powder (Fig. 5). However, the baseline level of the peak area associated with CFZ F I diffraction did not decrease as rapidly as the CFZ FNP dispersion was digested, and the sporadic spikes in intensity associated with drawing particles of crystalline CFZ into the capillary continued throughout the whole 30 min that the IF containing CFZ FNP powder was digested. This indicated that whilst the overall level of dispersed crystalline CFZ in the formulations was decreasing, there was still a considerable amount of crystalline drug remaining in the CFZ API formulation, whilst most of the crystalline material was removed by digestion of the CFZ API formulation. As previously reported (Salim et al., 2019a), some residual crystalline CFZ was observed when these digestions were performed using the traditional large-volume apparatus and so this result was not entirely unexpected. The potential reasons for this will be elaborated on in the discussion section.

## 4 Discussion

### 4.1 Optimising the small-volume digestion vessel

In the small-volume digestion vessel the pH microelectrode was positioned around 5 mm above the magnetic micro-stirrer bar. This was ideal to immerse the pH microelectrode tip with an adequate distance from the stirrer bar to avoid disrupting stirring of the digesting formulations. The legs of the 3D-printed vial holder plates (Fig. 2) were designed such that the top plate held the pH probe at this height (Fig. 3). The stirring speed was kept as high as possible in an attempt to maintain a fine suspension of drug powder, provide adequate mixing of the digest and the water (at 37 °C) surrounding the 20 mL vial (Taylor, 1985). Once digestions were completed, the 20 mL vial acting as the digestion vessel could be readily exchanged with a new vial containing pre-prepared dispersions for the next digestion, reducing the time required for cleaning the sample environment between measurements. The 3D-printed vial holder itself was cheap to produce and could be rescaled to accommodate different vial sizes and beakers.

### 4.2 Practical issues encountered in performing small-volume digestion experiments

Whilst no statistically significant differences were observed between the titration profiles for a given emulsion digested in either the small- or large-volume apparatus, there was substantially greater uncertainty in the amount of titrant added in the small-volume digestions during back titration to pH 9 at the end of each digestion. Digestion of the infant formula leads to the formation of calcium soaps of the fatty acids generated during digestion, and this can lead to solids collecting near the bottom of the vial that adhere to the stirrer bar. Given that the vortex generated by the stirrer bar in the 20 mL scintillation vial with vertical walls is not as strong as that in the traditional large-volume apparatus with tapered walls and conical shape, it was found that there was a greater tendency for calcium soaps to accumulate on the stirrer bar, making it stickier and slowing stirring of the mixture. This in turn may result in the amount of titrant added during the back-titration stage being greater in the small-volume apparatus than the large-volume apparatus, if the titrant is added more rapidly than the pH around the pH probe can adequately equilibrate. This issue is of course exacerbated by the precipitation of more calcium soaps as the pH of the digested emulsion is increased.

A recent inter-laboratory review of *in vitro* digestions using the INFOGEST protocol compared the measured lipolytic activity of different batches of enzyme in vessels with different shapes amongst other experimental factors (Grundy et al., 2021). This study found that whether the shape of the digestion vessel was conical (as in the large-volume digestion vessel) or cylindrical (as in the small-volume digestion vessel) was a statistically significant source of variation in lipolytic activities determined by pH-stat titration. Of all of the measurement parameters tested, conical vessels displayed the lowest coefficients of variation between activity measurements and cylindrical vessels the greatest. This difference was attributed to better mixing in the conical vessels and is consistent with the coefficients of variation observed in the conical and cylindrical digestion vessels used in this work (Table S8, electronic supporting information).

Adequate mixing of the sample within the digestion vessel and adequate wetting of drug particles is an important issue in the context of the *in situ* SAXS measurements of drug dissolution. Whilst it was previously reported that most CFZ API diffraction and essentially all of the CFZ FNP diffraction disappeared during digestion in 3.8% fat IF formulations in the large-volume digestion vessel (Salim et al., 2019a), this was not the case in the small-volume digestion vessel of this work. Diffraction from CFZ F I crystals was observed sporadically throughout the digestion of the CFZ FNP formulation and in the initial stages of the CFZ API formulation. This could be attributed to multiple factors including CFZ crystals becoming stuck in the tubing leading to the capillary clamped in the X-ray beam and therefore not being returned to the digesting infant formula and inadequate mixing and/or wetting of the sample in the vial leading to residual crystalline CFZ in the digesting emulsion that was sampled periodically. As an additional complication, as titrant was added, the meniscus of the dispersion rose further up the vial and any floating crystals not mixed with the bulk dispersion would therefore rise further from the opening of the tubing leading to the SAXS sampling capillary if mixing were inadequate. These issues are largely avoidable with the traditional large-volume apparatus as multiple/larger stirrer bars can be added to generate a stronger vortex promoted by the conical shape of the vessel as already discussed. Furthermore, the continuous flow of sample through the tubing connected to the capillary effectively washes out the capillary throughout the measurements, preventing the accumulation of undissolved drug in the tubing unless the tubing itself becomes blocked by viscous digest material or precipitated calcium soaps. Nonetheless, the small-volume experimental configuration replicated the results of previous larger volume studies whilst providing an opportunity to conserve resources during early-stage development of lipid-based formulations containing clofazimine FNPs.

Therefore, whilst it is possible to measure the dissolution of crystalline drug during digestion using this small-volume digestion apparatus using push-pull sampling, further improvements to the system are required to provide adequate mixing, and these are most likely to be obtained by changing the shape of the sample vessel to allow the formation of a more effective vortex as in the traditional large-volume digestion apparatus. The issue of drug crystals adhering to the walls of the sampling tubing is more difficult to address as there is insufficient volume of sample in the cell for flow-through sampling and reducing the internal diameter of the sampling tubing to remove a smaller flow through volume increases the likelihood of drug crystals blocking the tubing. The use of a robotic sampling system in conjunction with the cell to allow for rinsing of the capillary and tubing between diffraction acquisitions may also be explored in future experiments.

## 5 Conclusions

A miniaturised lipolysis apparatus was designed that allowed *in vitro* digestion measurements on 5 mL digests to be coupled to synchrotron SAXS. A 3D-printed vial holder was used to mount all components required for pH monitoring, fatty acid titration and sampling in a synchrotron X-ray beam, which could be easily modified to suit other digestion vessels. In the configuration studied, a minimum sample volume of 4.2 mL was required to maintain pH control. There were no statistically significant differences between the relative amount of fatty acids titrated in the small-volume and large-volume digestion vessels for any of the formulations tested. However, the uncertainties in the amounts of fatty acids titrated were greater when using the small-volume digestion apparatus, and this was attributed to less effective mixing of the samples, particularly during the back-titration to pH 9 at the end of the measurements. Whilst the previously reported trend for a reduction in the amount of dispersed CFZ crystals in infant formula during digestion was replicated during small-volume experiments coupled to synchrotron SAXS, there was evidence of crystalline material either in the dispersion or adhered to the sampling tubing and these are aspects of the small-volume digestion apparatus, again attributed to less effective mixing, that require future improvement. The 3D-printed sample holder provides a versatile platform for adapting the shape and size of the digestion vessel used. Further development of such small-volume digestion vessels offers reduced consumption of material for pre-clinical *in vitro* studies on the digestibility of lipid-based formulations and drug solubilisation therein when a limited supply of either drug or formulation lipids is available.

## CRediT authorship contribution statement

**Nafia F. Khan:** Investigation, Methodology, Writing – original draft, Writing – review & editing. **Malinda Salim:** Conceptualization, Investigation, Methodology, Writing – original draft, Writing – review & editing. **Syaza Y. Binte Abu Bakar:** Investigation, Methodology, Writing – review & editing. **Kurt Ristroph:** Investigation, Methodology, Writing – review & editing. **Robert K. Prud'homme:** Writing – review & editing, Supervision. **Adrian Hawley:** Conceptualization, Methodology, Writing – review & editing. **Ben J. Boyd:** Conceptualization, Investigation, Methodology, Writing – original draft, Writing – review & editing, Supervision. **Andrew J. Clulow:** Conceptualization, Investigation, Methodology, Writing – original draft, Writing – review & editing, Supervision.

## Declaration of Competing Interest

The authors declare the following financial interests/personal relationships which may be considered as potential competing interests:

Andrew Clulow reports a relationship with Australian Research Council that includes: funding grants.

## Appendix A Supplementary data

Supplementary material includes nutritional information for the milk and infant formula used in the study; dimensions of the printed sample holder; dynamic light scattering results; statistical comparison and fitting parameters for the titration results; UV-visible spectrometry standard curve for clofazimine; images of drug dispersions and lipolysis apparatus; comparative titration profiles between small and large volume apparatus. Image 1
