# Supplementary material for: Small-volume in vitro lipid digestion measurements for assessing drug dissolution in lipid-based formulations using SAXS
Source: Int J Pharm X. 2022 Feb 9;4:100113. doi: 10.1016/j.ijpx.2022.100113 (PMC8881665; doi:10.1016/j.ijpx.2022.100113)
Supplement: Supplementary file 1 — Supplementary material includes nutritional information for the milk and infant formula used in the study; dimensions of the printed sample holder; dynamic light scattering results; statistical comparison and fitting parameters for the titration results; UV-visible spectrometry standard curve for clofazimine; images of drug dispersions and lipolysis apparatus; comparative titration profiles between small and large volume apparatus. [file mmc1.pdf]

Supporting Information for Small-volume *In Vitro* Lipid Digestion Measurements for Assessing Drug Dissolution in Lipid-based Formulations using SAXS

Nafia F. Khan[1,2], Malinda Salim[2], Syaza Y. Binte Abu Bakar[2], Kurt Ristroph[3], Robert K. Prud'homme[3], Adrian Hawley[4], Ben J. Boyd,[*,2,5] and Andrew J. Clulow[*,2,4]

[1]*UCL School of Pharmacy, University College London, 29-39 Brunswick Square, London WC1N 1AX, UK*

[2]*Drug Delivery, Disposition and Dynamics, Monash Institute of Pharmaceutical Sciences, Monash University (Parkville Campus), 381 Royal Parade, Parkville, Victoria 3052, Australia*

[3] *Department of Chemical and Biological Engineering, Princeton University, Princeton, New Jersey 08544, United States*

[4]*Australian Synchrotron, ANSTO, 800 Blackburn Road, Clayton, Vicoria 3168, Australia*

[5]*Department of Pharmacy, University of Copenhagen, Universitetsparken 2, 2100 Copenhagen, Denmark*

Corresponding author details

Prof. Ben J. Boyd – Drug Delivery, Disposition and Dynamics, Monash Institute of Pharmaceutical Sciences, 381 Royal Parade, Parkville, Victoria 3052, Australia. tel: +61399039720, email: ben.boyd@monash.edu

Dr Andrew J. Clulow – BioSAXS beamline, Australian Synchrotron, ANSTO, 800 Blackburn Road, Clayton, Victoria 3168, Australia. tel: +61385404175, email: clulowa@ansto.gov.au.

Table S1 A comparison of nutritional information for bovine milk (3.8% fat) and the infant formula (IF) powder prepared (dispersed) used in this study.

**Nutritional Information**	**Milk (per 100 mL)**	**IF prepared (per 100 mL)**
Total fat	3.8 g	3.8 g
Saturated fat	2.5 g	2.1 g
Protein	3.4 g	1.5 g
Carbohydrate	4.8 g	7.9 g
Sodium	40 mg	36 mg
Calcium	115 mg	60 mg
Vitamin A	41 µg	60 µg
Riboflavin (Vitamin B2)	0.2 mg	0.1 mg

Table S2 Dimensions for vial holding chamber used on Sketchup 3D software.

**Layer**	**Component**	**Dimensions (mm)**
**Top**	Leg height	32
	Leg radius	4.5
	Platform thickness	2
	Platform outer radius	30
	Thermocouple/water circulation hole radius	1
	Inner hole radius	3 (with ~5 mm extensions in a cross shape to allow access for tubing)
	Leg hole radius	5.5
**Middle**	Leg height	18
	Leg radius	4.5
	Platform thickness	2
	Platform outer radius	30
	Thermocouple/water circulation hole radius	1
	Inner hole radius	14.5
	Leg hole radius	29
**Base**	Leg height	9
	Leg radius	4.5
	Platform thickness	2
	Platform outer radius	30
	Thermocouple/water circulation hole radius	1
	Inner hole radius	N/A
	Leg hole radius	5.5

Note: Middle layer printed four times to create a stack of the right height such that the top layer was above the top of the vial.

Table S3 Particle sizing by dynamic light scattering (DLS) for clofazimine (CFZ) FNPs showing nanoscale size distribution range and PDI <0.5, averaged from a stock solution of CFZ 5 mg/mL in 10 mL THF and 5 mg/mL lecithin.

**Parameter**	**Before lyophilisation**	**After lyophilisation and reconstitution**
Z-average diameter (nm)	74.7	2230
PDI	0.198	0.638
Attenuator (average)	8.94	3.00

Table S4 Statistical comparisons of the amounts of fatty acids titrated during the digestion of the different IF/drug formulations tested in the small-volume digestion apparatus. p values were derived by one-way ANOVA with Tukey post hoc (n = 3) comparing total FFAs titrated (with back-titration corrections applied). No statistically significant differences ($p < 0.05$) were observed and the formulations are denoted as follows: Infant formula only = IF; IF + halofantrine = Hf; IF + clofazimine API = CFZ API; and IF + CFZ flash-nanoparticles = CFZ FNP.

**Comparison**	**p-values at given digestion time (small-volume digestions)**						
	1 min	2 mins	4 mins	8 mins	16 mins	32 mins	55 mins
IF / CFZ API	0.371	0.972	1.000	1.000	0.994	0.988	0.966
IF / CFZ FNP	0.758	0.977	1.000	1.000	0.999	0.997	0.986
IF / Hf	1.000	0.999	0.963	0.900	0.776	0.718	0.592
CFZ API / CFZ FNP	0.996	1.000	1.000	1.000	1.000	1.000	1.000
CFZ API / Hf	0.568	0.805	0.917	0.981	0.991	0.990	0.986
CFZ FNP / Hf	0.914	0.822	0.800	0.925	0.961	0.968	0.967

Table S5 Statistical comparisons of the amounts of fatty acids titrated during the digestion of the different IF/drug formulations tested in the large-volume digestion apparatus. p values were derived by one-way ANOVA with Tukey post hoc (n = 3) comparing total FFAs titrated (with back-titration corrections applied). Formulations are denoted as above in Table S4.

**Comparison**	**p-values at given digestion time (large-volume digestions)**						
	1 min	2 mins	4 mins	8 mins	16 mins	32 mins	55 mins
IF / CFZ API	0.957	1.000	1.000	1.000	1.000	1.000	0.999
IF / CFZ FNP	0.927	1.000	1.000	1.000	1.000	1.000	1.000
IF / Hf	0.993	0.935	0.962	0.975	0.983	0.995	0.995
CFZ API / CFZ FNP	1.000	1.000	1.000	1.000	1.000	1.000	1.000
CFZ API / Hf	0.621	0.813	0.969	0.997	1.000	1.000	1.000
CFZ FNP / Hf	0.546	0.743	0.913	0.979	0.995	1.000	1.000

Table S6 Statistical comparisons of the amounts of fatty acids titrated during the digestion of the different IF/drug formulations tested in the small- and large-volume digestion (amount of fatty acids titrated divided by 4) apparatus. p values were derived by one-way ANOVA with Tukey post hoc (n = 3) comparing total FFAs titrated (with back-titration corrections applied). Formulations are denoted as above in Table S4.

**Comparison**	**p-values at given digestion time**						
	1 min	2 mins	4 mins	8 mins	16 mins	32 mins	55 mins
IF small / large	1.000	1.000	1.000	0.994	0.970	0.921	0.881
CFZ API small / large	0.999	0.984	0.914	0.804	0.752	0.618	0.606
CFZ FNP small / large	1.000	0.992	0.894	0.789	0.765	0.620	0.655
Hf small / large	0.805	0.400	0.247	0.134	0.170	0.315	0.521

Table S7 Linear fitting parameters for the titration data between 1 and 32 mins in Figure 4 [amount of fatty acids released versus $\log_2$(time)].

**Formulation**	**Gradient (mmol)**		**Pearson Correlation Coefficient**	
	**Small-volume**	**Large-volume**	**Small-volume**	**Large-volume**
**IF**	0.177 ± 0.056	0.453 ± 0.072	0.929	0.961
**CFZ API**	0.116 ± 0.039	0.319 ± 0.042	0.947	0.984
**CFZ FNP**	0.132 ± 0.043	0.344 ± 0.017	0.967	0.986
**Hf**	0.105 ± 0.016	0.356 ± 0.067	0.979	0.991

Table S8 Comparison of the total volumes of titrant added during the forward (titration during digestion at pH 7.5) and back titrations (addition of sodium hydroxide at the end of the digestions to raise the pH to 9) for each of the formulations tested. Titrant = 0.5 M NaOH. For the average titrated volumes, the coefficient of variation (standard deviation/mean) is given in parentheses.

**Formulation**	**Digestion Vessel Size**	**Forward Titration Volume (mL)**	**Average Forward Titration Volume (mL)**	**Back Titration Volume (mL)**	**Average Back Titration Volume (mL)**
IF	Small	0.409	0.409 ± 0.024 ($\sigma/\mu = 0.06$)	1.674	1.748 ± 0.980 ($\sigma/\mu = 0.56$)
IF	Small	0.386		0.807	
IF	Small	0.434		2.763	
IF	Large	1.593	1.595 ± 0.029 ($\sigma/\mu = 0.02$)	3.498	4.133 ± 0.888 ($\sigma/\mu = 0.21$)
IF	Large	1.568		3.753	
IF	Large	1.625		5.147	
CFZ API	Small	0.375	0.436 ± 0.063 ($\sigma/\mu = 0.14$)	1.010	1.365 ± 0.507 ($\sigma/\mu = 0.37$)
CFZ API	Small	0.434		1.139	
CFZ API	Small	0.500		1.946	
CFZ API	Large	1.614	1.645 ± 0.027 ($\sigma/\mu = 0.16$)	3.033	3.344 ± 0.391 ($\sigma/\mu = 0.12$)
CFZ API	Large	1.662		3.783	
CFZ API	Large	1.658		3.217	
CFZ FNP	Small	0.500	0.408 ± 0.083 ($\sigma/\mu = 0.20$)	1.946	1.452 ± 0.497 ($\sigma/\mu = 0.34$)
CFZ FNP	Small	0.386		1.460	
CFZ FNP	Small	0.339		0.951	
CFZ FNP	Large	1.554	1.587 ± 0.057 ($\sigma/\mu = 0.04$)	3.836	3.737 ± 0.126 ($\sigma/\mu = 0.03$)
CFZ FNP	Large	1.653		3.779	
CFZ FNP	Large	1.553		3.595	

Hf	Small	0.430	0.447 ± 0.032	0.864	0.997 ± 0.190
Hf	Small	0.428	(σ/μ = 0.07)	1.215	(σ/μ = 0.19)
Hf	Small	0.483		0.913	
Hf	Large	1.824	1.734 ± 0.078	3.338	2.914 ± 0.377
Hf	Large	1.699	(σ/μ = 0.04)	2.784	(σ/μ = 0.13)
Hf	Large	1.680		2.619	

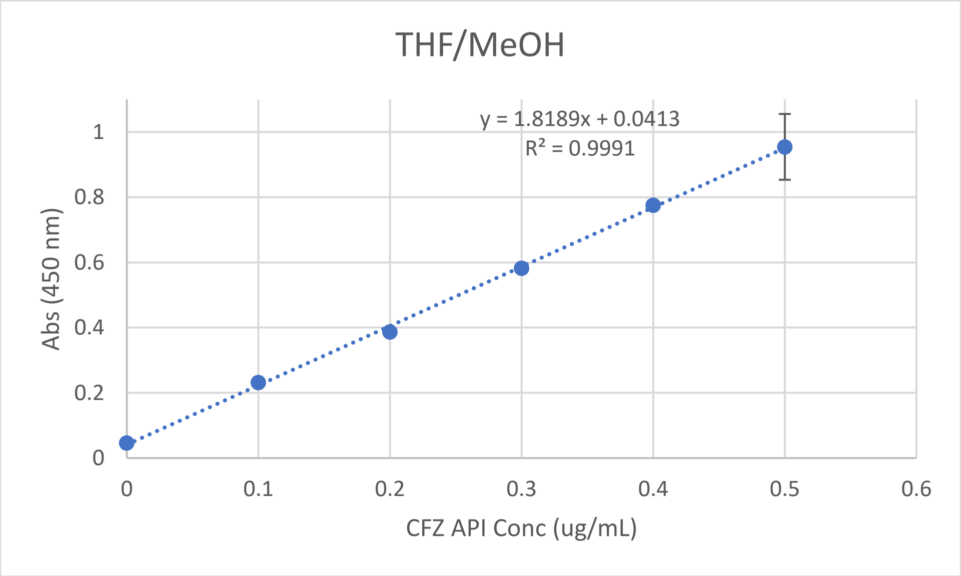

Figure S1 Clofazimine calibration curve in tetrahydrofuran (THF) and methanol (MeOH).

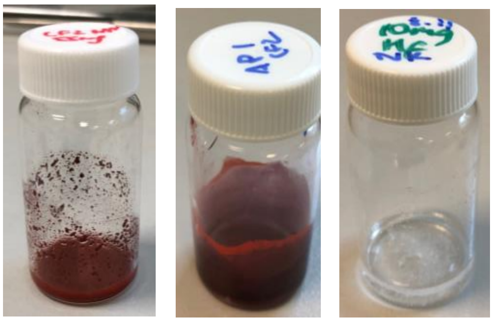

Figure S2 From L to R: Clofazimine (CFZ) flash-nanoprecipitated particles (FNP), CFZ API and Halofantrine (Hf) after acidifying with 0.01 M HCl to represent gastric conditions.

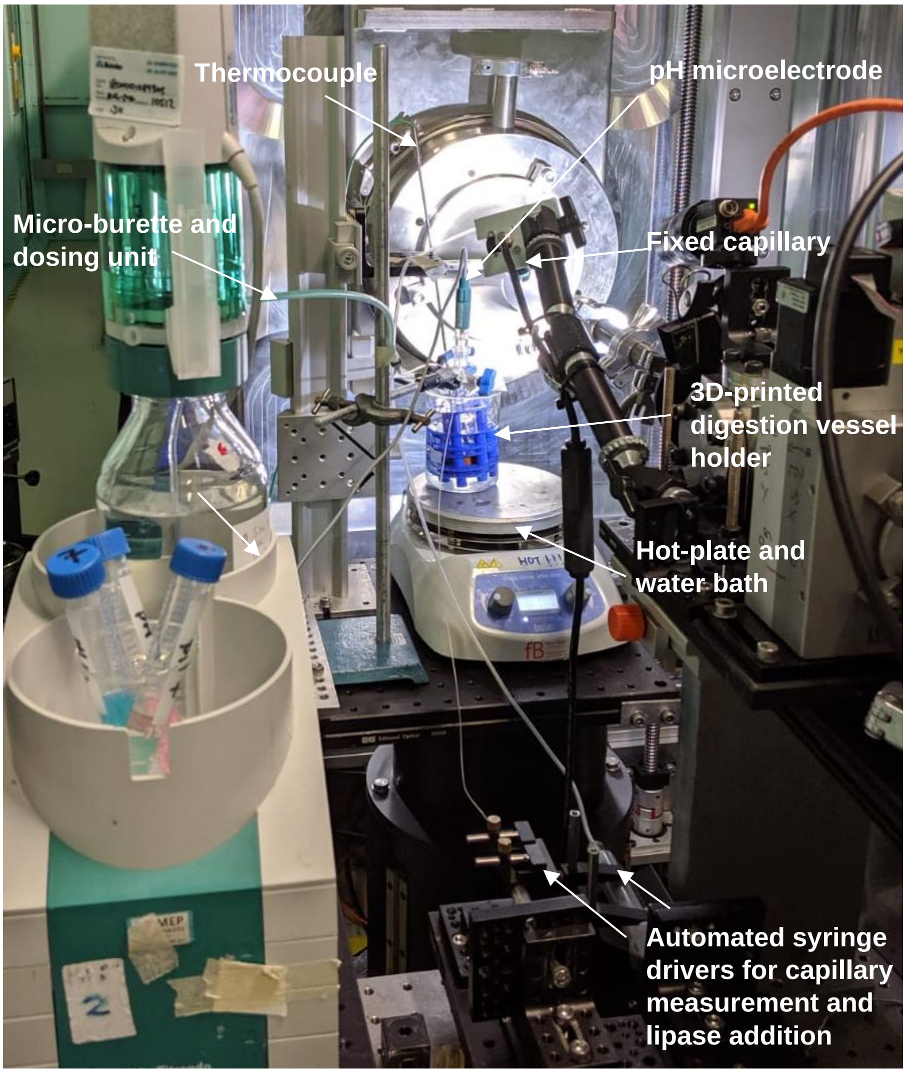

Figure S3 Miniaturised in vitro lipolysis apparatus installed on the SAXS/WAXS beamline at the Australian Synchrotron (ANSTO, Clayton, Victoria). Not shown: connected PC using Tiamo 3.2 software. Photograph by AJC.

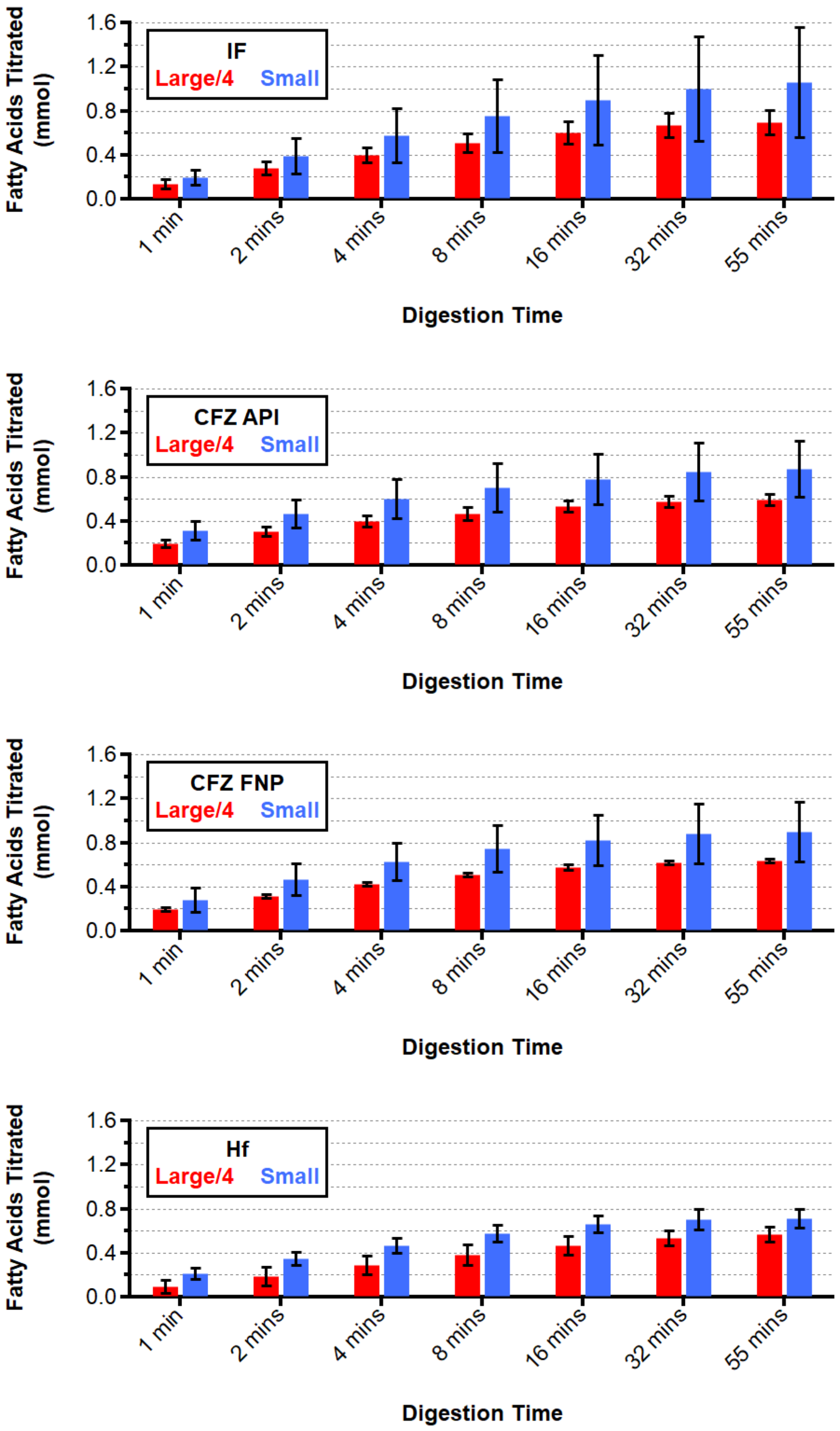

Figure S4 Comparison of the titration profiles for each formulation and the differences between small- and large-volume experiments (mean ± standard deviation, n = 3). The amount of fatty acids titrated in

the large-volume digestion apparatus has been divided by 4 for direct comparison. All plots are on the same scale.
